# Paracoccidioidomycosis in the spine: case report and review of the literature: Paracoccidioidomicose na coluna vertebral: relato de caso e revisão da literatura

**DOI:** 10.1590/1516-3180.2015.02691801

**Published:** 2015-04-14

**Authors:** José Alexandre Lopes da Silva Alvarenga, Délio Eulálio Martins, Michel Kanas, Hugo Gustavo Kunzle Elizeche, Adriana Macêdo Dell'Aquila, Eloy De Avila Fernandes, Marcelo Wajchenberg, Eduardo Barros Puertas

**Affiliations:** 1 MD. Spine Resident, Department of Orthopedics and Traumatology, Universidade Federal de São Paulo (Unifesp), São Paulo, SP, Brazil.; 2 MD, PhD. Attending Physician, Department of Orthopedics and Traumatology, Universidade Federal de São Paulo (Unifesp), São Paulo, SP, Brazil.; 3 MD. Spine Fellow, Department of Orthopedics and Traumatology, Universidade Federal de São Paulo (Unifesp), São Paulo, SP, Brazil.; 4 MD, PhD. Infectious Disease Specialist, Department of Orthopedics and Traumatology, Universidade Federal de São Paulo (Unifesp), São Paulo, SP, Brazil.; 5 MD, PhD. Affiliated Professor, Department of Imaging Diagnostics, Universidade Federal de São Paulo (Unifesp), São Paulo, SP, Brazil.; 6 MD, PhD. Affiliated Professor, Department of Orthopedics and Traumatology, Universidade Federal de São Paulo (Unifesp), São Paulo, SP, Brazil.; 7 MD, PhD. Full Professor, Department of Orthopedics and Traumatology, Universidade Federal de São Paulo (Unifesp), São Paulo, SP, Brazil.

**Keywords:** Paracoccidioides, Paracoccidioidomycosis, Spine, Discitis, Osteomyelitis

## Abstract

**CONTEXT:**

Paracoccidioidomycosis is a systemic form of mycosis that spreads hematogenously, secondarily to reactivation of lung infection or infection at another site or to new exposure to the causative agent. Few cases of bone involvement have been reported in the literature and involvement of the spine is extremely rare.

**CASE REPORT:**

We describe a case of a 68-year-old male patient with spondylodiscitis at the levels L4-L5 caused by presence of the fungus *Paracoccidioides brasiliensis,* which was diagnosed through percutaneous biopsy. The patient was treated with sulfamethoxazole and trimethoprim for 36 months, with complete resolution of the symptoms.

**CONCLUSION:**

Spondylodiscitis caused by the fungus *Paracoccidioides brasiliensis* is uncommon. However, in patients with chronic low-back pain who live or used to live in endemic regions, this infection should be considered as a possible differential diagnosis.

## RESUMO

**CONTEXTO:**

Paracoccidioidomicose é uma micose sistêmica de disseminação hematogênica, secundária a reativação de uma infecção pulmonar ou de outro sítio, ou a uma nova exposição ao agente causador. Poucos casos de envolvimento ósseo são relatados na literatura, e o acometimento da coluna vertebral é extremamente raro.

**RELATO DE CASO:**

Descrevemos o caso de um paciente masculino de 68 anos, apresentando espondilodiscite no nível L4-L5, causada pela presença do fungo *Paracoccidioides brasiliensis,* diagnosticada após biópsia percutânea. O paciente foi tratado com sulfametoxazol e trimetoprim por 36 semanas, com resolução completa dos sintomas.

**CONCLUSÃO:**

A espondilodiscite causada pelo fungo *Paracoccidioides brasiliensis* é incomum, mas, em pacientes portadores de lombalgia crônica que viveram ou vivem em regiões endêmicas, deve ser considerada como um possível diagnóstico diferencial.

## INTRODUCTION

Paracoccidioidomycosis or South American blastomycosis is a granulomatous systemic disease caused by the fungus *Paracoccidioides brasiliensis* .^1^^,^^2^ It presents geographic distribution limited to Latin America, with higher incidence observed in Brazil, where its prevalence and clinical and epidemiological characteristics vary according to the region of this country.^2^^,^^3^^,^^4^ Contamination occurs through inhalation of the fungus, which is followed by lymphatic or hematogenous spreading. The infective process is usually asymptomatic and tends to come to an end spontaneously, but remaining residual lesions may contain viable fungus for years.^5^ The most commonly affected areas are the skin, mucous membranes and lungs. However, bone involvement is unusual and involvement of the spine is rare.^4^^,^^6^^,^^7^

## CASE REPORT

A 68-year-old male patient, a former farm laborer, presented with low-back pain that had started four months earlier. Over the preceding two years, he had had three episodes of pneumonia and lost 20 kg. He had systemic arterial hypertension, which was controlled through use of antihypertensive drugs. There was no diabetes, thyroid diseases or any other metabolic cause for weight loss. He was a smoker (50 pack-years) and moderate alcohol user.

The symptoms became worse during trunk flexion and there was painful low-back muscle palpation. There were no abnormalities on neurological examination, no fever and no night pain. No signs of consumptive syndrome were noted.

Laboratory tests revealed increased levels of C-reactive protein (CRP) of 118.5 mg/dl (normal, 0.05 mg/dl) and erythrocyte sedimentation rate (ESR) of 53 mm/h (normal < 15 mm/h). The white blood cell count was 6-7 k/mm^3^ (normal, 4.0-11.0 k/mm^3)^. A sputum culture was negative for tuberculosis. Chest radiography and computed tomography (CT) scan did not reveal any signs of tumor or infection.

**Figure 1:**
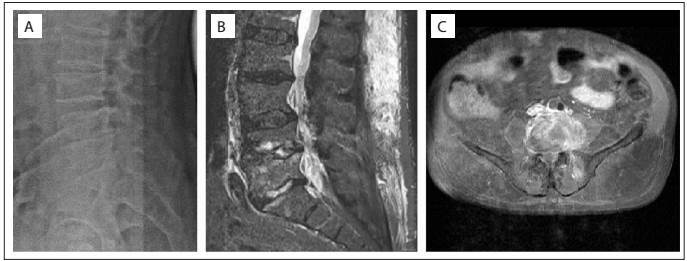
Radiography and magnetic resonance imaging (MRI) of lumbosacral spine. (A) Lateral radiograph showing irregular vertebral plates of L4 and L5, and reduction of disc spaces L4-L5 and L5-S1. (B) MRI in sagittal view with T1 fat-saturation contrast, showing spondylodiscitis of L4-L5, with subligamentous inflammatory tissue and reduced disc height of L4-L5 and L5-S1. (C) MRI in axial view with T1 gadolinium contrast, showing inflammatory tissue involving L4 and left psoas muscle.

Radiography of the lumbosacral spine showed diffuse degenerative changes, irregular vertebral endplates of L4 and L5, and reduction of disc spaces L4-L5 and L5-S1 (Figure 1A).

Lumbar spine magnetic resonance imaging (MRI) demonstrated spondylodiscitis of L4-L5, with subligamentous abscess anterior to the vertebral bodies, reduced disc height of L4-L5 and L5-S1 and spinal stenosis from L2 to S1 (Figures 1B and ^1C^).

**Figure 2:**
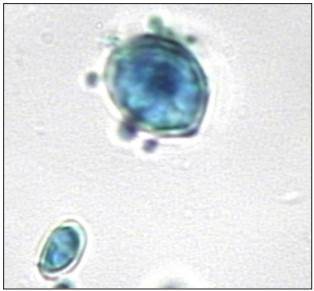
Histological slide of L4, stained with lactophenol cotton blue, showing fungal spores of *Paracoccidioides brasiliensis* (400 X).

After analyzing MRI data, main hypotheses for this case were the presence of a tumor, tuberculosis or pyogenic spondylodiscitis. However, transpedicular biopsy of L4 revealed infection with *Paracoccidioides brasiliensis* (Figure 2).

Since the patient was not presenting any mechanical or neurological instability, clinical treatment was implemented. Therapy with itraconazole (200 mg/day) was started, but the patient presented an adverse reaction to this drug (worsening of liver function), and therefore this was replaced with sulfamethoxazole and trimethoprim (20 mg/kg) for the rest of the treatment. After 36 months of treatment with sulfamethoxazole and trimethoprim (20 mg/kg), the patient became asymptomatic and the inflammatory blood tests (CRP and ESR) returned to normal.

**Figure 3:**
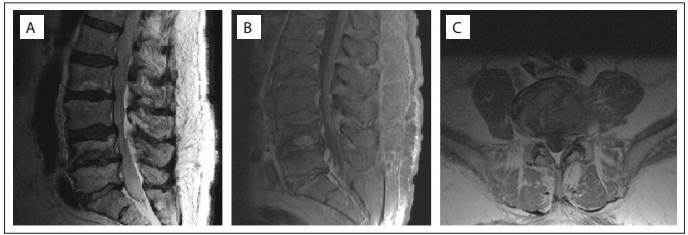
Magnetic resonance imaging (MRI) after 36 months of antibiotic therapy showing reduction of paraspinal soft-tissue edema and abscess. (A) Lateral T2 view. (B) Lateral T1 gadolinium view. (C) Axial T1 gadolinium view.

A control radiograph showed ligament ossification of L4-L5, subchondral sclerosis and reduction of disc space. Control MRI demonstrated reduction of edema and abscess in the paraspinal soft tissues (Figure 3).

## DISCUSSION

Paracoccidioidomycosis was originally described in 1908 by Lutz.^8^ The infection is caused by inhaling particles of the dimorphic fungus *Paracoccidioides brasiliensis* .

The major risk factor for acquiring this infection are activities involving close contact with soil, given that the soil is contaminated with this fungus in endemic areas. It is primarily acquired during the first two decades of life, with peak incidence between the ages of 10 and 20 years.^4^^,^^5^^,^^7^

Individuals who become infected may develop two main clinical forms: the acute or subacute form (juvenile type), with severe involvement of internal organs and mononuclear phagocyte system; or the chronic form (adult type), which represents 90% of the cases, with insidious evolution and involvement of the lungs that can leave residual damage (latent foci) with fungus that remains viable for years. In the unifocal presentation, the mycosis is restricted to one organ, which is rare. Most cases affect multiple organs simultaneously.^4^^,^^9^

Once the fungus has become established in the body, it can affect any organ or tissue, but most commonly it affects the skin, mucous membranes and lungs.^4^^,^^6^^,^^7^ Osteoarticular involvement is unusual, with an incidence that ranges from 5.9 to 23%. This is generally associated with lung infection that spreads to bones through lympho-hematogenous dissemination.^4^^,^^7^^,^^10^^,^^11^^,^^12^^,^^13^

Chest wall bones are the ones most affected by the disease, including ribs, sternum, scapula and acromion.^13^ Spinal involvement is rare.

A search in the main electronic databases (PubMed, Embase and Lilacs) using the key words: "Paracoccidioidomycosis" and "Spine" was conducted (Table 1). Only four case reports were found,^7^^,^^14^^,^^15^^,^^16^ and only one of them described infection in the vertebral body. The three other reports comprised one of sternal infection,^14^ one of spinal cord blastomycotic granuloma^15^ and one of vertebral infection caused by *Blastomyces dermatitidis* .^16^

**Table 1:**
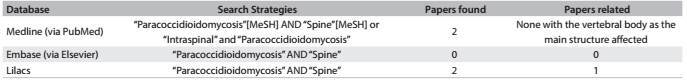
Database search results for Paracoccidioidomycosis and Spine on December 29, 2015

Our case is the first in the literature to show the MRI characteristics of the vertebral body during infection by *Paracoccidioides brasiliensis,* and after antibiotic treatment.

The diagnosis can be made by isolating and identifying the fungus; by direct mycological examination of sputum; by histopathological, cytopathological or cytological examination after puncture biopsy or culturing; or through serological techniques.^17^

In this study, the patient presented the clinical and epidemiological characteristics of the disease, which included: male gender, farm laborer in an endemic area and age over 40 years.^6^^,^^7^^,^^8^^,^^9^^,^^10^^,^^11^^,^^12^^,^^13^^,^^17^^,^^18^^,^^19^ The patient needed to have become infected during his second decade of life, when he worked in the fields for 10 years in an endemic area of the disease. After more than 40 years of incubation, the focus was reactivated.

Fungal infections in the spine are rare, and generally occur in patients with an impaired immune system.^20^ The infectious agent usually reaches the vertebral body through small metaphyseal arteries arising from larger periosteal arteries that are branches of spinal arteries. Another proposed route is retrograde flow of venous blood via the Batson plexus.^21^^,^^22^

In this case, we could not detect the way in which the infection arrived at the vertebral body (arterial or venous route). However, the patient's medical history of three recent bouts of pneumonia and his weight loss, smoking and alcohol use were likely to have contributed towards compromising his immune system and making him susceptible to reactivation of latent fungal infection.

It was not possible to confirm that the previous repeated episodes of pneumonia were manifestations of paracoccidioidomycosis, since no yeast cells were isolated previously. Although chest radiography and CT scan did not show any signs of infection or scars, the patient did not present any other episode of pneumonia after the antibiotic treatment for spinal infection, over the subsequent two-year follow-up period.

After 36 months, the patient was seen to be clinically asymptomatic, and his blood inflammatory markers were within the normal range. These parameters are considered to be the most important healing criteria.^14^

Control radiographs showed L4-L5 ankylosis, subchondral sclerosis and reduction of disc space. MRI showed regression of paraspinal soft-tissue edema and abscess. We considered that the better-quality imaging provided by MRI, was important for certifying remission of the infection^14^ (Figure 3).

## CONCLUSION

Spondylodiscitis caused by the fungus *Paracoccidioides brasiliensis* is uncommon, but in patients with chronic low-back pain who live or used to live in endemic regions, this infection should be included as a possible diagnosis. In the case presented, the patient had a satisfactory evolution with appropriate treatment.
